# Supplementary material for: Methods and considerations for estimating parameters in biophysically detailed neural models with simulation based inference
Source: bioRxiv. 2023 Apr 17:2023.04.17.537118. Preprint. [Version 1] doi: 10.1101/2023.04.17.537118 (PMC10153146; doi:10.1101/2023.04.17.537118)
Supplement: Supplement 1 [file NIHPP2023.04.17.537118v1-supplement-1.pdf]

bioRxiv preprint doi: https://doi.org/10.1101/2023.04.17.537118; this version posted April 17, 2023. The copyright holder for this preprint (which was not certified by peer review) is the author/funder, who has granted bioRxiv a license to display the preprint in perpetuity. It is made available under aCC-BY 4.0 International license.

Supporting information

Prior distribution setup and sampling

Prior samples $\theta_i \in \mathbb{R}^d$ were generated by using the PyTorch Uniform distribution on the interval [0,1). The values in each dimension were then linearly mapped to the range of their corresponding parameter values. For parameters specifying the maximum conductance $\bar{g}$ (nanosiemens, nS) of synaptic connections in HNN, the values were additionally exponentiated in base 10 after being mapped to the appropriate range.

Table 1. Simulation parameters and SBI training

**RC Circuit**	**Range**	**Transform**
Amplitude 1 (mA)	(0, 1)	linear
Amplitude 2 (mA)	(0, 1)	linear
Latency (ms)	(-75, 75)	linear
**HNN "RC"**	**Range**	**Transform**
Distal Exc (nS)	(1e-4, 1e-3)	exponential
Proximal Exc (nS)	(1e-4, 1e-3)	exponential
Latency (ms)	(-75, 75)	linear
**ERPs**	**Range**	**Transform**
Distal Exc (nS)	(1e-5, 1)	exponential
Proximal Exc (nS)	(1e-5, 1)	exponential
Distal Inh (nS)	(1e-5, 1)	exponential
Proximal Inh (nS)	(1e-5, 1)	exponential
**Beta Events**	**Range**	**Transform**
Distal Var (ms^2)	(0, 20)	linear
Proximal Var (ms^2)	(0, 40)	linear

The prior support and transform function for the parameters of examples shown in the text.

bioRxiv preprint doi: https://doi.org/10.1101/2023.04.17.537118; this version posted April 17, 2023. The copyright holder for this preprint (which was not certified by peer review) is the author/funder, who has granted bioRxiv a license to display the preprint in perpetuity. It is made available under aCC-BY 4.0 International license.

Simulation and SBI training

Prior samples and simulations were all generated and stored in the form of `NumPy` binary arrays before neural density estimator training. The `SBI` Python package was used for all neural density estimator training and posterior evaluation. A masked autoregressive flow architecture was utilized for approximation of the posterior distribution. Posteriors for all examples were trained using a dataset of 100,000 samples from the prior distribution. Gaussian white noise was added to training observations x_i [54]. The variance of the Gaussian noise added to observations was 0.01 for RC circuit simulations, and 1e-5 for HNN simulations.

All analysis was performed on the Expanse supercomputing cluster managed by XSEDE and the Neuroscience Gateway. HNN simulations were generated using the Dask distributed scheduler configured for the SLURM workload manager.

Diagnostic heatmaps were constructed by defining a grid over the support of the prior with a range of $[0.05, 0.95]^d$, with a resolution of 10 samples in each dimension d.
